# A Novel Prognostic Model Based on Autophagy-Related Long Non-Coding RNAs for Clear Cell Renal Cell Carcinoma

**DOI:** 10.3389/fonc.2021.711736

**Published:** 2021-08-03

**Authors:** Xinyuan Li, Haitao Yu, Zongjie Wei, Xin Gou, Simin Liang, Fu Liu

**Affiliations:** ^1^Department of Urology, The First Affiliated Hospital of Chongqing Medical University, Chongqing, China; ^2^Chongqing Key Laboratory of Molecular Oncology and Epigenetics, Chongqing, China

**Keywords:** ccRCC, autophagy-related genes, long non-coding RNAs, risk model, prognosis

## Abstract

**Background:**

Renal cell carcinoma (RCC) is one of the most common malignant tumors of the urinary system, of which the clear cell renal cell carcinoma (ccRCC) accounts for the most subtypes. The increasing discoveries of abundant autophagy-related long non-coding RNAs (ARLNRs) lead to a resurgent interest in evaluating their potential on prognosis prediction. Based on a large number of ccRCC gene samples from TCGA and clinics, ARLNRs analysis will provide a novel perspective into this field.

**Methods:**

We calculated the autophagy scores of each sample according to the expression levels of autophagy-related genes (ARGs) and screened the survival-related ARLNRs (sARLNRs) of ccRCC patients by Cox regression analysis. The high-risk group and the low-risk group were distinguished by the median score of the autophagy-related risk score (ARRS) model. The functional annotations were detected by gene set enrichment analysis (GSEA) and principal component analysis (PCA). The expression levels of two kinds of sARLNRs in the renal tumor and adjacent normal tissues and cell lines were verified.

**Results:**

There were 146 ARLNRs selected by Pearson analysis. A total of 30 sARLNRs were remarkably correlated with the clinical outcomes of ccRCC patients. Eleven sARLNRs (AC002553.1, AC092611.2, AL360181.2, AP002807.1, AC098484.1, AL513218.1, AC008735.2, MHENCR, AC020907.4, AC011462.4, and AC008870.2) with the highest prognosis value were recruited to establish the ARRS in which the overall survival (OS) in the high-risk group was shorter than that in the low-risk group. ARRS could be treated as an independent prognostic factor and has significant correlations with OS. The distributions of autophagy genes were different between the high-risk group and the low-risk group. In addition, we also found that the expression levels of AC098484.1 in ccRCC cell lines and tumor tissues were lower than those in HK-2 and adjacent normal tissues, but AL513218.1 showed the inverse level. Furthermore, the AC098484.1 expressed decreasingly with the more advanced T-stages, but AL513218.1 gradually increased.

**Conclusion:**

Our study identified and verified some sARLNRs with clinical significances and revealed their potential values on predicting prognoses of ccRCC patients, which may provide a novel perspective for autophagy-related research and clinical decisions.

## Introduction

With the approximately 295,000 new cases and 134,000 deaths worldwide per year, renal cell carcinoma (RCC) accounts for approximately 3% of adult malignancies, of which clear cell RCC (ccRCC) is the most common histological subtype (1, 2). It is well known that ccRCC is not sensitive to chemotherapy and radiotherapy, especially in the advanced ccRCC (3, 4). Hence, a series of explorations of replacement therapies such as targeted therapy, anti-angiogenic therapy, and immunotherapy have been motivated. With the rapid development of bioinformatics analysis technology, a growing number of attentions have been focused on the values of the risk characteristics including clinical and molecular features on the prognosis assessment of cancer patients (5, 6). Although accumulated risk factors including glycolysis-related gene characteristics (7, 8), autophagy-related gene characteristics (9, 10), and long noncoding RNAs (lncRNAs)-related characteristics (11, 12) have been identified to show satisfactory results on prediction outcomes of patients with various malignancies including RCC, discovering more novel evaluating methods to help make clinical decisions still received enormous interest.

Autophagy is a lysosomal degradation pathway in the cellular biology process, which plays a significant role on protecting cells and tissues from stressors in normal physiological processes (13, 14). Emerging reports also highlighted the crucial effects of autophagy on a variety of pathological processes of malignant tumors, so the deeper associations between autophagy, tumor characteristics, and clinical strategies including diagnosis, treatment, and follow-up remain anticipated. Autophagy-related genes (ARGs) have received wide attention because of their attractive and viable predicting values of certain malignancies (15, 16). Therefore, we attempt to discover whether novel and sensitive autophagy-related biomarkers can also provide the basis for making more personalized and appropriate clinical decisions of patients with ccRCC.

LncRNA, a class of more than 200 nucleotides in length and absenting of potential coding proteins, have been reported to be involved in the occurrence, development, and metastasis of tumors (17, 18). Additionally, a series of lncRNAs targeting ARGs and regulating autophagy have also been identified. Three lncRNAs (MBNL1-AS1, HAND2-AS1, and MIR100HG) were examined to predict the prognosis of gastric cancer patients (19). LncRNAs DNAH17-AS1 and RP11-400N13.2 can predict the prognosis of colorectal cancer patients (20). However, the potential values of autophagy-related lncRNAs (ARLNRs) on forecasting the prognosis of patients with ccRCC have been poorly studied.

Therefore, we designed this study to give a perspective into the clinical potential values of ARLNRs on prognosis prediction of ccRCC patients. We detected some ARLNRs in the ccRCC transcriptome database and clinical samples, combining their clinical features, to find the connections between ARLNRs and clinical outcomes of ccRCC patients. The present study focuses on the underlying mechanisms and effects of ARLNRs on prognosis of ccRCC, highlighting the notion that a novel and accurate predicting model actually elicits a broad spectrum of effects that provide foundations for making appropriate clinical strategies.

## Methods

### Clinical Renal Samples and Human Renal Cell Lines

We collected ccRCC and normal adjacent tissues of 186 patients who were diagnosed with ccRCC by the First Affiliated Hospital of Chongqing Medical University. Human normal renal cell line HK-2 and renal cancer cell lines (786-O, RCC-23, RCC-JF, and CAKI) were purchased from the American Type Culture Collection (Manassas, Virginia, USA). Cells were cultured by DMEM and 1640 medium, which were supplemented with 10% fetal bovine serum (FBS), 100 u/ml penicillin, and 100 mg/ml streptomycin (Gibco, Gaithersburg, MD, USA). Cells were incubated at 37°C in 5% CO_2_. The medium was changed every 1-3 days.

### Data Download and Pretreatment

We downloaded transcriptome RNA-sequences data of ccRCC samples that contained data from 72 nontumor samples and 539 ccRCC samples from the TCGA data portal (https://portal.gdc.cancer.gov/). Meanwhile, we downloaded and extracted the clinical data of these samples (the OS of patients ≤ 30 days were excluded because these patients probably died of unpredictable factors) (**Supplementary File 1**: **Table S1**). These data were currently updated in September 08, 2020. We combined RNA-sequences results into a matrix file and converted the Ensembl IDs of RNA into a matrix of gene symbols by Perl language (http://www.perl.org/). Next, we downloaded the gene biotype file (GRCh38) from NCBI and used the Perl language to distinguish between lncRNAs and mRNAs. The autophagy-related genes (ARGs) were assessed from the Human Autophagy Database (http://autophagy.lu/). Pearson correlation analysis was used to analyze the correlations between ARGs and the expression levels of lncRNAs in ccRCC patients. Autophagy-related long noncoding RNAs (ARLNRs) were identified by a standard of |r|>0.7 and *P*<0.001 (**Supplementary File 2**).

### Creation of the Autophagy-Related Risk Score Model

ARLNRs correlated with overall survival in ccRCC patients were confirmed as sARLNRs. sARLNRs were selected by univariate COX analysis (*P*<0.001). The protective and deleterious portion of sARLNRs was detected by the Hazard ratio (HR). Multivariate analysis was used to screen sARLNRs to establish the IRRS model (*P <*0.05). In order to further detect the relationships between sARLNRs and clinical features, we created an ARRS model to divide ccRCC patients into the low-risk group and the high-risk group. The ARRS model was established by the expression data multiplied by Cox regression coefficients. The formula was as follows: [Expression levels of AC002553.1 * (-0.40913)] + [Expression levels of AC092611.2 * (-0.19811)] + [Expression levels of AL360181.2 * (0.12365)] + [Expression levels of AP002807.1 * (0.23705)] + [Expression levels of AC098484.1 * (-0.37844)] + [Expression levels of AL513218.1 * (0.54416)] + [Expression levels of AC008735.2 * (-0.21448)] + [Expression levels of MHENCR * (-0.09323)] + [Expression levels of AC020907.4 * (0.32531)] + [Expression levels of AC011462.4 * (0.13916)] + [Expression levels of AC008870.2 * (0.86763)]. Patients were divided into high-risk and low-risk groups based on the median risk score.

### Real-Time Quantitative PCR

Triazole (TaKaRa) was used to extract the RNA from ccRCC cell lines and clinical tissues according to the manufacturer’s instructions. cDNA Synthesis Kit (TaKaRa) and RNA (1μg) were utilized to reverse transcribed cDNA. The quantitative polymerase chain reaction (qPCR) was run on an ABI 7500 real-time PCR system (Applied Biosystems) by the SYBR-Green method (TaKaRa). Relative expression levels of lncRNAs normalized to β-actin were calculated by the 2^−ΔCt^ method. The primer sequences are shown in **Table 1**. There are three assays per cDNA sample.

**Table 1:** The primer sequences of AC098484.1 and AL513218.1.

**AC098484.1**	F primer (5’-3’)	TAATGTCTCTTCCATCCGGCTCT
	R primer (5’-3’)	ACTCCGATGATACTTGGTTGCT
**AL513218.1**	F primer (5’-3’)	CTCAGTGACCATCTGCACATC
	R primer (5’-3’)	CTCTGTCCCCTTAGTTACCAT
**β-actin**	F primer (5’-3’)	AAACGTGCTGCTGACCGAG
	R primer (5’-3’)	TAGCACAGCCTGGATAGCAAC

F primer, forward primer; R primer, reverse primer.

### Bioinformatics Analysis

Pearson correlation analysis and univariate Cox analysis were used to verify the sARLNRs. The ROC curves were drawn using the survival ROC package of the R software. We used univariate and multivariate Cox analysis to identify the independent prognostic factors for ccRCC patients. Principal component analysis (PCA) was used to display the autophagy expression levels of ccRCC samples, and gene set enrichment analysis (GSEA) was used to detect the different functional phenotype between the low‐risk group and the high‐risk group. Nomogram plot was used to evaluate the survival probabilities of ccRCC patients by the rms package of the R software. We displayed Pearson correlations analysis to detect the relationships between lncRNAs and mRNAs by limma package of the R software. Cytoscape software version 3.7.2 was used to demonstrate lncRNAs-mRNAs coexpression results.

## Statistical Analysis

All statistical analysis was conducted using the SPSS21.0 software (SPSS Inc, Chicago, IL) and GraphPad Prism8 (GraphPad Software Inc, La Jolla, CA). The clinical correlations were determined by ANOVA, post-hoc test (Boferroni method), and independent T-test. P<0.05 was considered to be of significant statistical difference.

## Results

### Acquisition of sARLNRs

**Figure 1:**
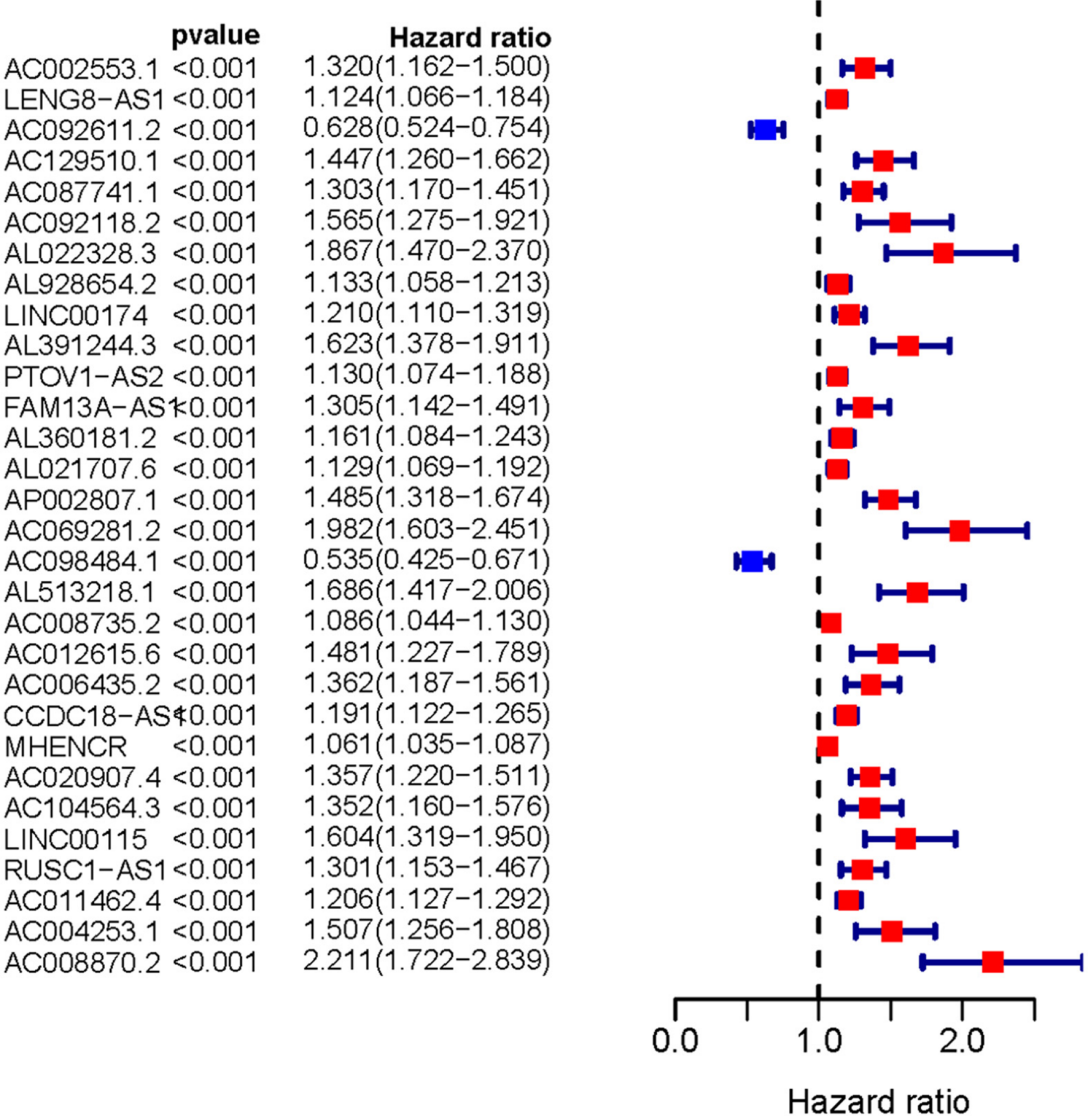
Survival-related ARLNRs forest plot. The hazard ratios of survival-related ARLNRs (AC002553.1, LENG8-AS1, AC092611.2, AC129510.1, AC087741.1, AC092118.2, AL022328.3, AL928654.2, LINC00174, AL391244.3, PTOV1-AS2, FAM13A-AS1, AL360181.2, AL021707.6, AP002807.1, AC069281.2, AC098484.1, AL513218.1, AC008735.2, AC012615.6, AC006435.2, CCDC18-AS1, MHENCR, AC020907.4, AC104564.3, LINC00115, RUSC1-AS1, AC011462.4, AC004253.1, and AC008870.2) were showed in the forest plot. Red parts represent upregulated sARLNRs, and green parts represent downregulated sARLNRs.

Transcriptome and clinical data of ccRCC samples were downloaded from the TCGA database. Next, the ensembl ID of ccRCC transcriptome data were converted into the gene name. We used the gene biotype file (GRCh38) from NCBI to distinguish between lncRNAs and mRNAs and used the Perl language. We screened 146 ARLNRs by Pearson correlation analysis. Based on univariate Cox regression analysis, we verified 30 ARLNRs correlated with overall survival (sARLNRs), including AC002553.1, LENG8-AS1, AC092611.2, AC129510.1, AC087741.1, AC092118.2, AL022328.3, AL928654.2, LINC00174, AL391244.3, PTOV1-AS2, FAM13A-AS1, AL360181.2, AL021707.6, AP002807.1, AC069281.2, AC098484.1, AL513218.1, AC008735.2, AC012615.6, AC006435.2, CCDC18-AS1, MHENCR, AC020907.4, AC104564.3, LINC00115, RUSC1-AS1, AC011462.4, AC004253.1, and AC008870.2. The forest map demonstrated the correlations between 30 sARLNRs and the hazard ratio clearly (**Figure 1**).

### Construction of the ARRS Model

**Figure 2:**
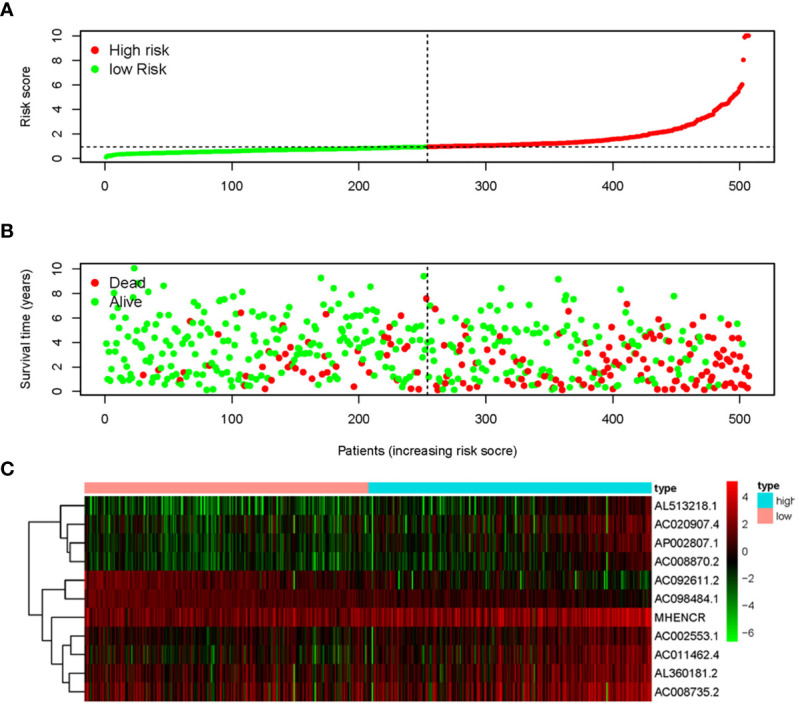
Autophagy-related risk score model (ARRS) was established by sARLNRs. The risk score distributions in the high-risk group and the low-risk group **(A)**. Survival status between the high-risk group and the low-risk group **(B)**. The heatmap of expression levels of sARLNRs **(C)**.

**Figure 3:**
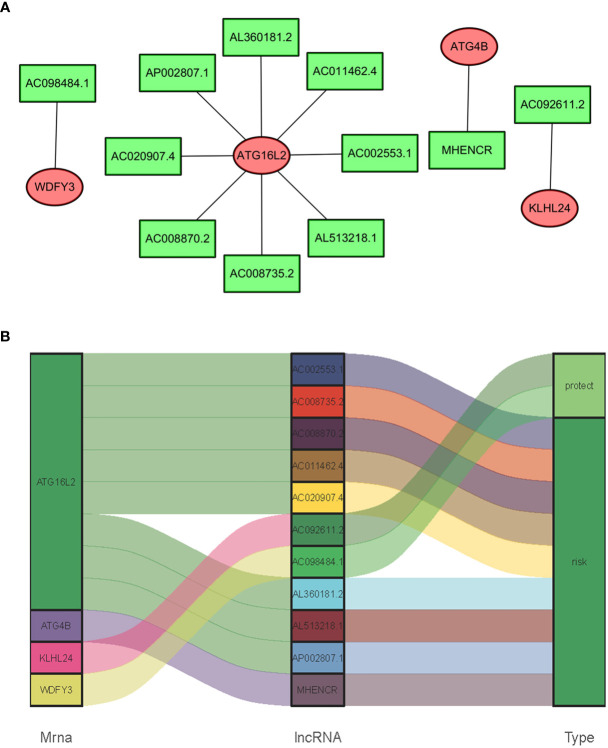
The ROC curve and survival curve of the ARRS model. The receiver operating characteristic (ROC) curve of the ARRS model **(A)**. Kaplan‐Meier survival curve of OS in ccRCC patients from the high-risk group and the low-risk group. The results illustrated that the high-risk group has poor prognosis **(B)**.

The 11 sARLNRs (AC002553.1, AC092611.2, AL360181.2, AP002807.1, AC098484.1, AL513218.1, AC008735.2, MHENCR, AC020907.4, AC011462.4, and AC008870.2) (p<0.05) among the 30 sARLNRs were used to establish the ARRS model, of which the patients with ccRCC were divided into the high-risk group and the low-risk group (**Figure 2A**). In order to detect the relationships between 11 sARLNRs and prognosis, we draw the survival curves of these sARLNRs. The results demonstrated that the higher expression levels of AC092611.2 and AC098484.1 were correlated with the poorer OS, while the higher expression levels of AC002553.1, AL360181.2, AP002807.1, AL513218.1, AC008735.2, MHENCR, AC020907.4, AC011462.4, and AC008870.2 were related with longer OS (**Supplementary File 3**: **Figure S1**). The mortality rate of the high-risk group was higher than that of the low-risk group (**Figure 2B**). With the increase in the risk score, the expression levels of AC002553.1, AL360181.2, AP002807.1, AL513218.1, AC008735.2, MHENCR, AC020907.4, AC011462.4, and AC008870.2 were elevated, but the expression levels of AC092611.2 and AC098484.1 were decreased (**Figure 2C**). The AUC of 5 years ROC curve of the ARRS model was 0.733 (**Figure 3A**). The OS of the low-risk group was remarkably longer than that of the high-risk group (**Figure 3B**).

### The Relationships of sARLNRs and ARGs

**Figure 4:**
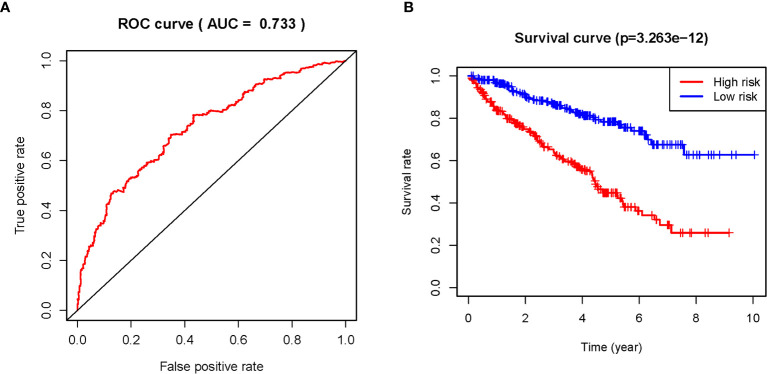
**(A, B)** The relationships between ARGs and sARLNRs. A Sankey diagram was used to visualize the relationships between lncRNAs, mRNAs, and risk factors.

In consideration of the fact that sARLNRs and ARGs can influence the occurrence, development, and progression of cancer, the lncRNAs-mRNAs coexpression relationship network was established by the Cytoscape software (**Figures 4A, B**). There was a significant correlation between 11 sARLNRs and 4 ARGs (r > 0.7 p < 0.001). The Sankey diagram was drawn to demonstrate the co-occurrences of sARLNRs and ARGs. The results showed that ATG16L2 in autophagy-related mRNAs may be the main components. In addition, AC092611.2 and AC098484.1 could be severed as the protective portion, while the rest of sARLNRs were the risk portion.

### The Correlations of Clinical Features and ARRS Model

**Figure 5:**
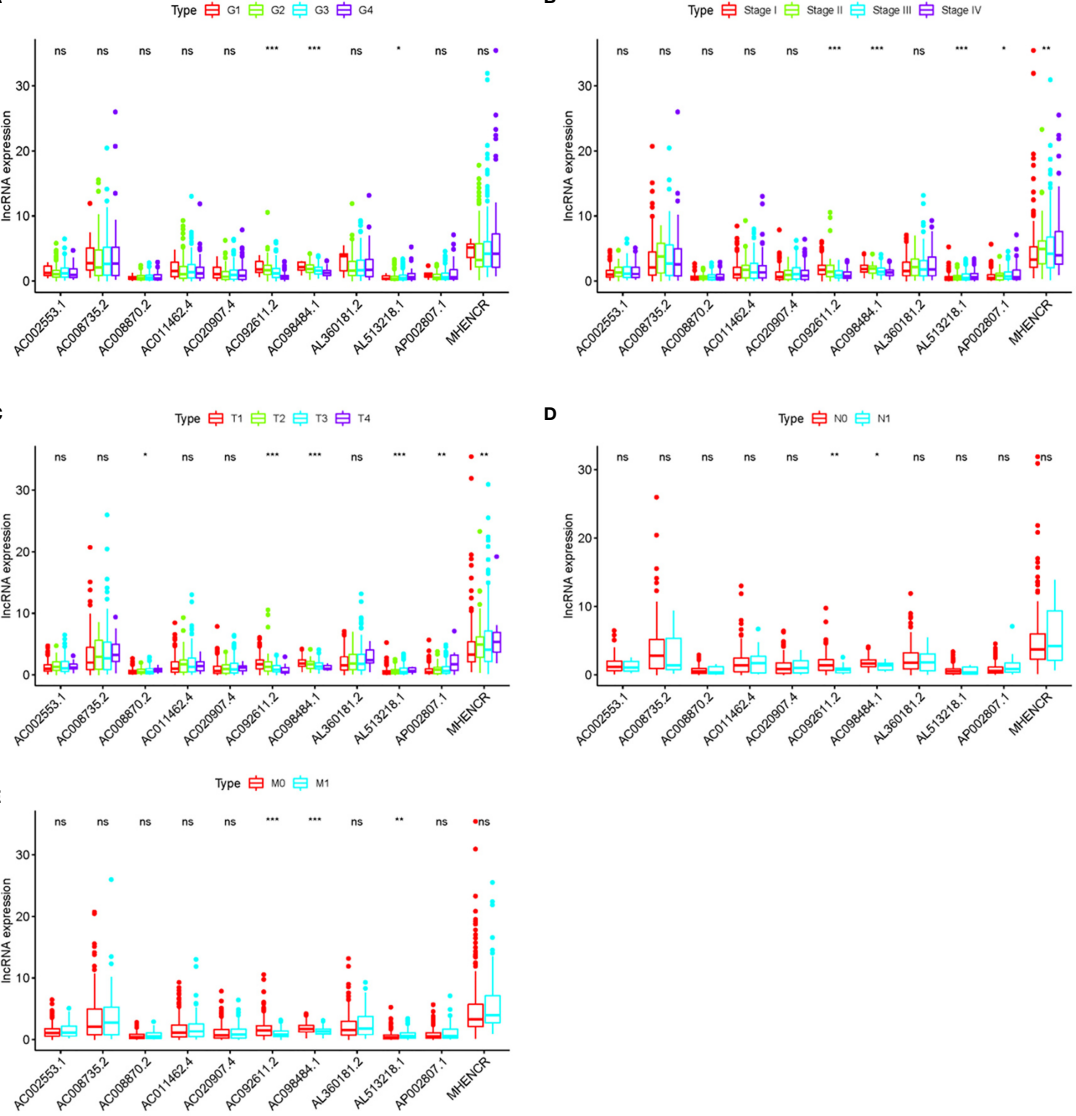
The relationships between the clinical features and sARLNRs. Relationships between 11 sARLNRs (AC002553.1, AC092611.2, AL360181.2, AP002807.1, AC098484.1, AL513218.1, AC008735.2, MHENCR, AC020907.4, AC011462.4, and AC008870.2) and clinical features were shown in **(A–E)**. The expression levels of AL513218.1 were decreased in the early stage, grade, T-stage, and M-stage; the expression levels of AP002807.1 and MHENCR were increased in the advanced stage and T-stage; the expression levels of AC008870.2 were enhanced in the advanced T-stage. (***p < 0.001; **p < 0.01; *p < 0.05; ns = p > 0.05).

**Figure 6:**
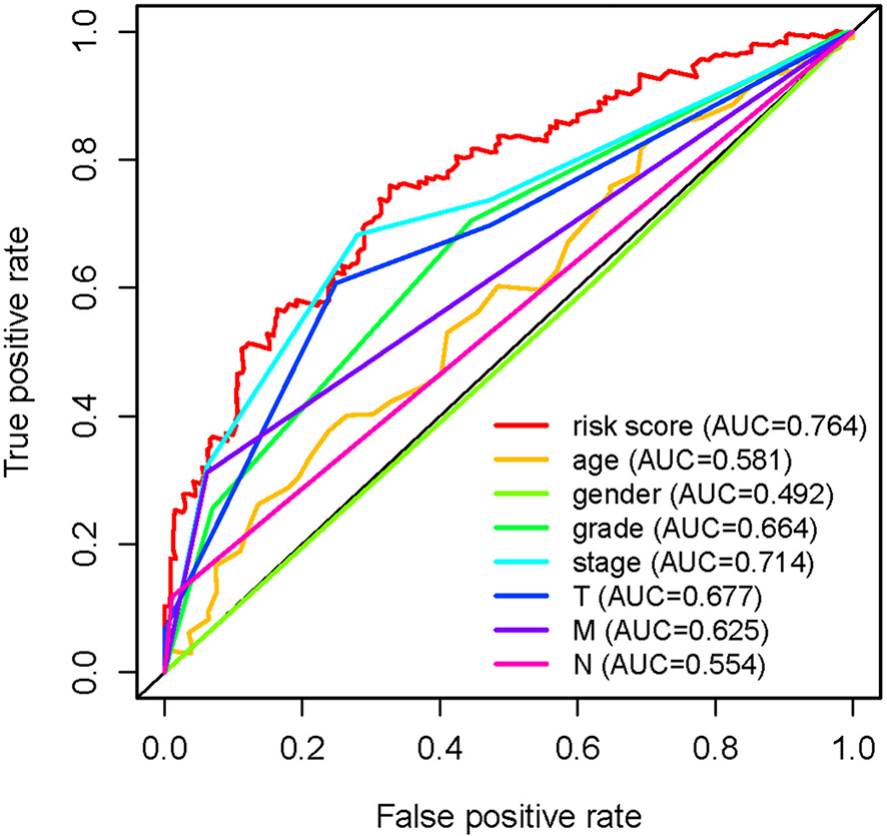
Multi-receiver operating characteristic (ROC) curves. The prognostic value of the independent prognosis factors was indicated by ROC curves.

In order to further detect the underlying clinical values of the ARRS model, we analyzed the relationships of ARRS and the clinical and demographic features, such as age, stage, grade, T-stage, N-stage and M-stage. We discovered that the expression levels of AC092611.2 and AC098484.1 were increased in the early stage, grade, T-stage, N-stage, and M-stage (**Figures 5A–E**); the expression levels of AL513218.1 were enhanced in the advanced stage, grade, T-stage, and M-stage; the expression levels of AP002807.1 and MHENCR were decreased in the early stage and T-stage; the expression levels of AC008870.2 were decreased in the early T-stage. Furthermore, we used Cox regressive analysis to detect whether the ARRS model can be served as the independent prognostic factor; the results showed age, grade, stage, T-stage, N-stage, M-stage, and ARRS were remarkably related with the prognosis of ccRCC patients in univariate Cox analysis (P<0.05). But in the multivariate Cox analysis, only age, grade, and ARRS were significantly correlated with the ccRCC patients’ prognosis (**Table 2**). The ROC curve represents the accuracy of the risk score model. The Area Under the Curve (AUC) of ARRS, age, gender, grade, stage, T-stage, N-stage, and M-stage are 0.764, 0.581, 0.492, 0.664, 0.714, 0.677, 0.625, and 0.554, respectively (**Figure 6**). These results suggested that the ARRS was a reliable independent prognostic factor.

**Table 2:** Univariate and multivariate COX analysis of ccRCC.

Variables	Univariate analysis				Multivariate analysis			P value
	HR	HR 95% low	HR 95% high	P value	HR	HR 95% low	HR 95% high	
**Age**	1.019	1.001	1.037	0.031	1.033	1.013	1.054	0.001
**Gender**	1.073	0.700	1.645	0.745	1.459	0.909	2.342	0.117
**Grade**	2.257	1.687	3.018	4.101e-08	1.475	1.047	2.078	0.025
**Stage**	1.898	1.566	2.301	6.391e-11	1.301	0.780	2.171	0.312
**T-stage**	1.977	1.559	2.508	1.866e-08	1.047	0.654	1.675	0.848
**M-stage**	4.262	2.749	6.608	9.129e-11	1.869	0.831	4.206	0.130
**N-stage**	3.035	1.568	5.873	0.001	1.362	0.647	2.868	0.415
**Risk score**	1.316	1.214	1.427	2.319e-11	1.214	1.104	1.334	5.656e-05

HR, Hazard Ratio.

**Figure 7:**
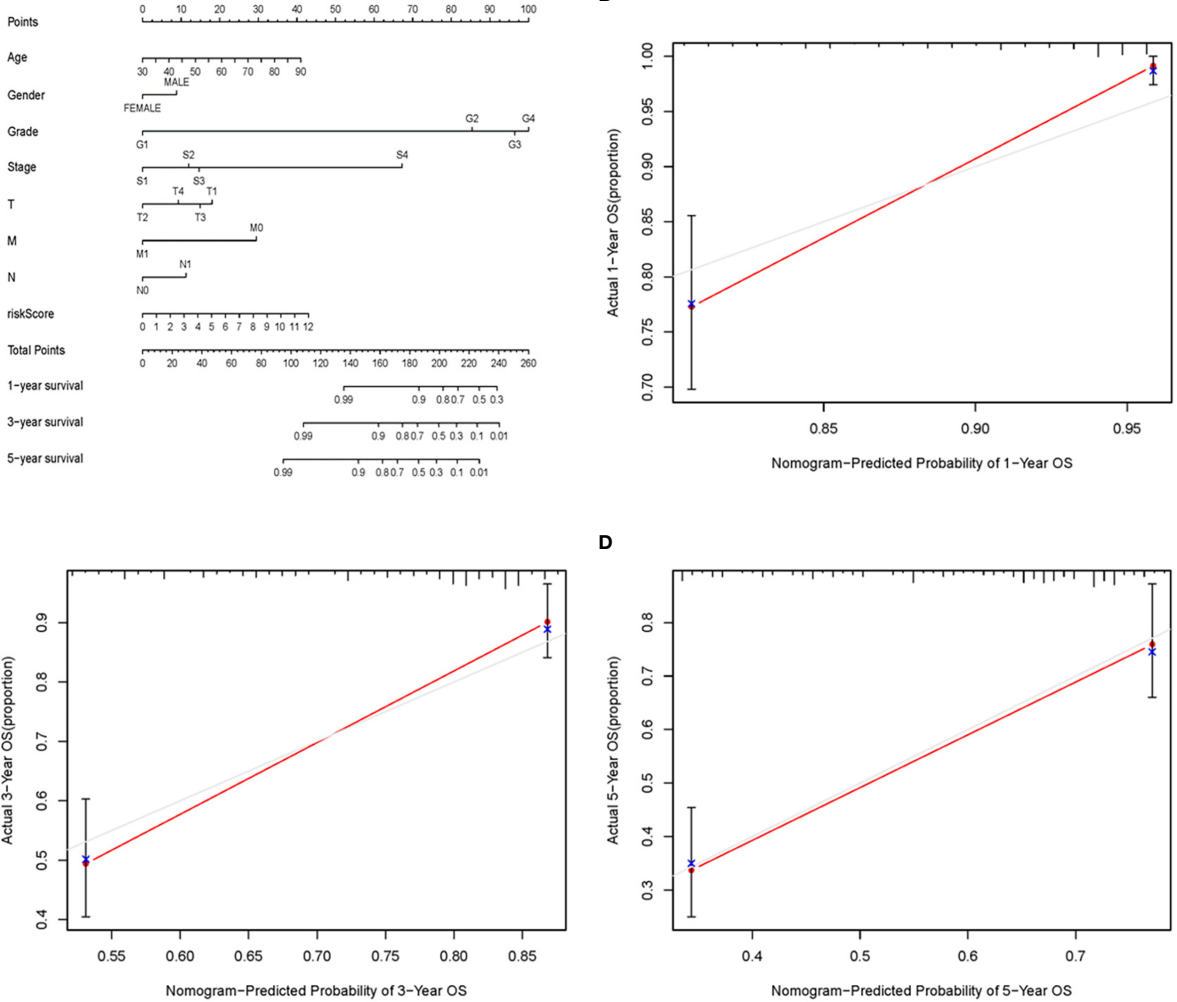
The nomogram and calibration curves of ARRS. Nomogram was used for predicting 1-, 3-, and 5-year survival probability of ccRCC patients **(A)**. The calibration curves of nomogram of 1 **(B)**, 3 **(C)**, and 5 **(D)** years.

To further detect the clinical application of ARRS, we established a nomogram of ccRCC patients by using the multivariate Cox analysis of clinical features and risk score (**Figure 7A**). We normalized the points of each ccRCC patient to a distribution of 0 to 100. We could forecast the survival probability of ccRCC patients in 1, 3, and 5 years by drawing a vertical line from the total points axis to each prognosis axis. The calibration curve of the nomogram of 1, 3, and 5 years showed that the nomogram would be a new reliable and accurate method for doctors to predict the prognosis of ccRCC patients (**Figures 7B–D**).

### Analysis of the Autophagy Status of the High- and Low-Risk Groups

**Figure 8:**
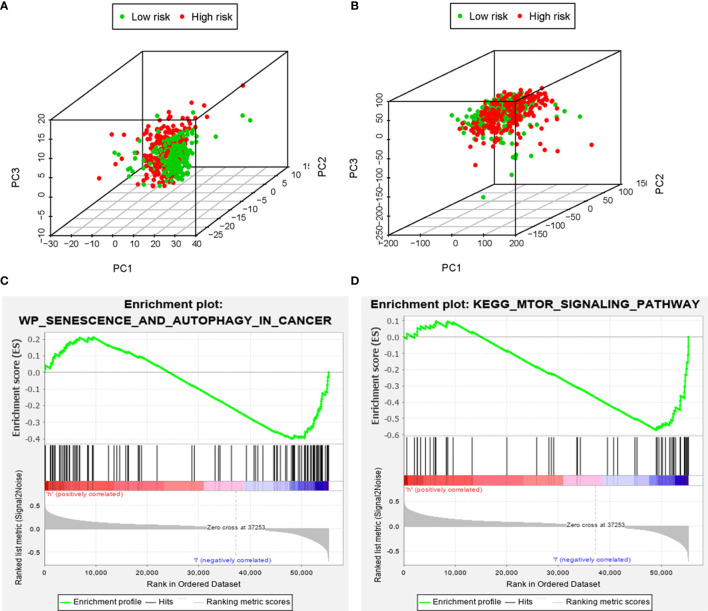
The principal components analysis (PCA) and gene set enrichment analysis (GSEA). The high‐risk group and the low‐risk group tended to express different autophagy status. PCA among the high‐risk group and the low‐risk group based on the autophagy‐related gene sets **(A)**. PCA among the high‐risk group and the low‐risk group based on the whole protein‐coding gene sets **(B)**. GSEA implied remarkable enrichments of autophagy in cancer and autophagy-related pathway (MTOR) in the high‐risk group **(C, D)**.

Based on the genome-wide expression sets and the ARG sets, we used the principal component analysis (PCA) to discover the different autophagy distribution patterns between the high-risk group and the low-risk group. The high-risk group and the low-risk group were separated into two parts of which the low-risk group had lower autophagy scores than the high-risk group in the ARG sets (**Figure 8A**). On the other hand, we could not find the separation of the high and low-risk groups by the genome-wide expression sets (**Figure 8B**). According to the results of GSEA, the low-risk group had the negative correlations of the MTOR signaling pathway and autophagy in cancer (p<0.05) (**Figures 8C, D**).

### AC098484.1 Was Downregulated but AL513218.1 Was Overexpressed, Especially in Patients With Advanced T-Stages

**Figure 9:**
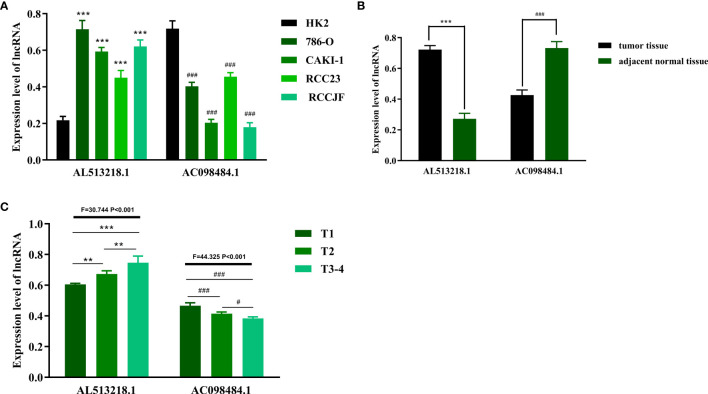
The expression levels of AL513218.1 and AC098484.1 *in vitro* and *in vivo.* The expression levels of AL513218.1 and AC098484.1 in renal cancer cell lines and renal tubular epithelial cell were measured by RT-qPCR **(A)**. ^***^ and ^###^ represent the significant difference compared with HK2 (P < 0.001). The RT-qPCR results of AL513218.1 and AC098484.1 in carcinoma tissues and adjacent normal tissues **(B)**. ^***^ and ^###^ represent the obvious difference compared with adjacent normal tissues (P < 0.001). The expression levels of AL513218.1 and AC098484.1 in various ccRCC tissues with different T-stages **(C)**. ^***^, ^**^, ^###^ and ^#^ represent the remarkable difference compared with another group (P < 0.001, P < 0.01, P < 0.001 and P < 0.05), respectively.

In hopes of further verifying the clinical values of the ARRS model, the focus next turned toward the detections of some sARLNRs involved in the model in various samples *in vitro* and *in vivo*. The results of RT-qPCR showed that the expression level of AC098484.1 in ccRCC cell lines (786O, CAKI-1, RCC23, RCCJF) was remarkably lower than that in the renal tubular epithelial cell line (HK2), but AL513218.1 showed a reverse trend (**Figure 9A**). To investigate the associations of the sARLNRs with clinicopathologic characteristics, we examined the expression levels of AL513218.1 and AC098484.1 in ccRCC samples of various T-stages. As illustrated in **Figure 9B**, compared with the adjacent normal tissues, AC098484.1 expressed less in ccRCC tissues, and the decreasing trend was more significant in samples of more advanced T-stages (**Figure 9C**); however, AL513218.1 was upregulated in ccRCC tissues (**Figure 9B**), especially in more advanced T-stages (**Figure 9C**).

## Discussion

The significance of autophagy in occurrence, progression, and prognosis of tumors has inspired more explorations of the potentials of ARGs on identifying some autophagy-related biomarkers to predict the prognosis of various malignancies. Although a class of microRNAs, lncRNAs, and immune-related biomarkers has offered increasing options for clinic, the discovery of the predictive effects of sARLNRs on ccRCC is still in its infancy.

Accumulated evidence indicates that autophagy is a relatively conservative process in normal physiological processes, but the correlations between tumors and autophagy remain controversial (21, 22). The roles of autophagy are dynamic in different stages of tumorigenesis and development, with a possible inhibitor of occurrence of tumors at the early stage but an underlying pro-tumoral factor for invasion and metastasis at the late stage (23). Although certain autophagy-related mRNAs and miRNAs have been verified to predict the survival of ccRCC patients and a large number of risk score models based on differentially expressed ARGs and autophagy-related miRNAs have been established to forecast survival (24, 25), most reports indicated these ARGs, absence in the missions of encoding proteins; therefore, lncRNAs gain more specificity on evaluating tumor actual conditions than other types of biomarkers. Given the inherent advantages of lncRNAs on cancer biological processes and the remarkable autophagy correlation of ARLNRs, exploring their values on predicting the prognosis of patients with ccRCC is eagerly awaited.

In the present study, 611 ccRCC patients were selected in TCGA for lncRNAs; combining with ARGs screened in the Human Autophagy Database (http://autophagy.lu/), 146 ARLNRs were verified eventually. We found the relationships of the prognosis of patients with ccRCC and the expression levels of the 146 ARLNRs, of which 30 ARLNRs had remarkable correlations with OS. We further verified 11 sARLNRs to create a risk score model that had the potential ability to distinguish patients with ccRCC into the low-risk group and the high-risk group with the differences of OS by multivariate Cox analysis and the risk score model. We also found the relationships between ARGs and sARLNRs. As a result of the molecular heterogeneity, we further detected that the predicting value of the 11 sARLNRs can be served as independent of the traditional risk factor and molecular characteristics by univariate and multivariate Cox analysis. The nomogram and calibration curve results showed that ARRS could be a reliable method to predict the OS of ccRCC patients accurately. The principal component analysis (PCA) method was utilized to detect the differences between the low-risk group and the high-risk group by the genome-wide set and the ARG expression set. According to the ARG set, the low-risk group and the high-risk group tended to be divided into two parts, with the low-risk group having lower autophagy scores than the high-risk group. When PCA was analyzed based on the genome-wide expression set, the autophagy status of these groups showed no remarkable separation. To further identify the functional annotation, GSEA was employed, and we found the more abundant autophagy-related pathways and processes in the high-risk group, such as the MTOR signaling pathway and autophagy in cancer. These results indicated that the risk forecasting scores based on the 11 sARLNRs can contribute to verifying the high-risk patients from the ccRCC patients with the same clinical characteristics or molecular characteristics; hence choosing an appropriate and individualized therapeutic strategy.

The values of ATGs in the present study were also supported by other studies of different centers. Daniela et al. demonstrated the significant effect of a prognostic model consisting of WIPI1, BAG1, and PEX3 autophagy-related genes in melanoma (26). Besides, Gu et al. proposed autophagy-related prognostic signature (BCL2, BIRC5, EIF4EBP1, ERO1L, FOS, GAPDH, ITPR1, and VEGFA) for predicting the prognosis of breast cancer (27). The predicting value of 11 sARLNRs (U62317.4, LINC01016, LINC02166, C6orf99, LINC00992, BAIAP2-DT, AC245297.3, AC090912.1, Z68871.1, LINC00578, and LINC01871) was also identified in breast cancer (9). Luan et al. developed a risk score model based on 10 sARLNRs (PCBP1-AS1, TP53TG1, DHRS4-AS1, ZNF674-AS1, GABPB1-AS1, DDX11-AS1, SBF2-AS1, MIR4453HG, MAPKAPK5-AS1, and COX10-AS1) to forecast the prognosis of glioma cancer patients (28). In spite of the features and importance of some ARGs and LNRNAs on tumor occurrence, development, progression, and autophagy responses have been revealed in some cancers, the genome-wide and completed analysis to identify more accurate and sensitive ARLNRs, especially in forecasting prognosis, remains sparse. Therefore, we employed a lot of ccRCC patients willing to join in the present research to further enhance the persuasion of clinical evidences. The verification results illustrated that the specific sARLNRs in the ARRS model indeed can be served as individual molecular biomarkers to evaluate the infiltration of autophagy and forecast OS of ccRCC patients. Although certain lncRNAs that were identified in Xuan’s study overlapped with our model, the ARRS model in the present study was generally quite different from Xuan’s model (29). Firstly, we combined the basic experiments such as qPCR to verify the expression level and clinical significance of ARLNRs, which were enrolled in the ARRS model in high amounts of clinical samples. Thus, we ascertained the prominent differences between AC098484.1 and AL513218.1 in various T-stages. These results further increase the feasibility and credibility of the ARRS model by which evaluated the prognosis of ccRCC patients. Besides, in this study, we also identified and verified some different ARLNRs that were used to establish the ARRS model; hence, more ccRCC-related biomarkers were supplemented.

In spite of the fact that we detected the effects of some sARLNRs on predicting the prognosis of ccRCC patients and further verified the expression levels of AL513218.1 and AC098484.1 in tumor tissues and ccRCC cell lines, there remain some limitations. Firstly, the clinical application values of these sARLNRs remain undefined. Then, in addition to AL513218.1 and AC098484.1, other sARLNRs included in the ARRS model should also be detected. Thirdly, we did not split the data into training and testing sets. Additionally, further verifications of the involved sARLNRs and autophagy are insufficient. Finally, in this study, we have displayed GSEA to predict the autophagy-related pathways of these lncRNAs, but more experimental methods including western blot and transmission electron microscope should be utilized to detect autophagy-related protein expression and autophagic structures, and to better validate the autophagic relevance of ARLNRs.

## Conclusion

In conclusion, we analyzed and verified the significant roles of sARLNRs on predicting the clinical outcomes of ccRCC patients. Our results establish a sensitive and accurate risk score model to evaluate the outcomes of ccRCC patients, and provide a novel perspective into further studies of autophagy, LNRNAs, and ccRCC prognosis.

## Data Availability Statement

The original contributions presented in the study are included in the article/**Supplementary Material**. Further inquiries can be directed to the corresponding authors. 

## Ethics Statement

The studies involving human participants were reviewed and approved by The First Affiliated Hospital of Chongqing Medical University. The patients/participants provided their written informed consent to participate in this study.

## Author Contributions

SL, XL, and XG designed and directed the research. FL was responsible for the paper writing and data analysis. ZW collected the samples. HY verified the gene expression levels. All authors contributed to the article and approved the submitted version.

## Conflict of Interest

The authors declare that the research was conducted in the absence of any commercial or financial relationships that could be construed as a potential conflict of interest.

## Publisher’s Note

All claims expressed in this article are solely those of the authors and do not necessarily represent those of their affiliated organizations, or those of the publisher, the editors and the reviewers. Any product that may be evaluated in this article, or claim that may be made by its manufacturer, is not guaranteed or endorsed by the publisher.

## Supplementary Material

Supplementary Table 1 The clinical information of ccRCC samples in TCGA database.

Supplementary Data Sheet 1 The expression information of autophagy-related long non-coding RNAs.

Supplementary Figure 1 The Kaplan-Meier analysis of AC092611.2, AC098484.1, AC002553.1, AL360181.2, AP002807.1, AL513218.1, AC008735.2, MHENCR, AC020907.4, AC011462.4 and AC008870.2.

## Abbreviations

ARGs, autophagy-related genes; ARLNRs, autophagy-related long non-coding RNAs; ARRS, autophagy-related risk score model; ccRCC, clear cell renal cell carcinoma; FBS, fetal bovine serum; GSEA, gene set enrichment analysis; HR, Hazard ratio; lncRNAs, Long non-coding RNAs; OS, overall survival; PCA, principal component analysis; RCC, Renal cell carcinoma; sARLNRs, survival-related ARLNRs; TCGA, The Cancer Genome Atlas.
