# Supplementary material for: A Novel Prognostic Model Based on Autophagy-Related Long Non-Coding RNAs for Clear Cell Renal Cell Carcinoma
Source: Front Oncol. 2021 Aug 3;11:711736. doi: 10.3389/fonc.2021.711736 (PMC8370088; doi:10.3389/fonc.2021.711736)
Supplement: Supplementary Table 1 — The clinical information of ccRCC samples in TCGA database. [file DataSheet_1.docx]

**Supplementary table: The summary of clinical characteristics of patients with ccRCC**

| **Characteristics** | **TCGA** |
| --- | --- |
| Age(years) |  |
| ≤65 | 352 |
| >65 | 185 |
| Gender |  |
| Male | 346 |
| Female | 191 |
| Grade |  |
| G1 | 14 |
| G2 | 230 |
| G3 | 207 |
| G4 | 78 |
| Stage |  |
| SI | 269 |
| SII | 57 |
| SIII | 125 |
| SIV | 83 |
| T-stage |  |
| T1 | 275 |
| T2 | 69 |
| T3 | 182 |
| T4 | 11 |
| N-stage |  |
| N0 | 240 |
| N1 | 17 |
| M-stage |  |
| M0 | 426 |
| M1 | 79 |
