# Supplementary figures and images for: A Novel Prognostic Model Based on Autophagy-Related Long Non-Coding RNAs for Clear Cell Renal Cell Carcinoma
Source: Front Oncol. 2021 Aug 3;11:711736. doi: 10.3389/fonc.2021.711736 (PMC8370088; doi:10.3389/fonc.2021.711736)

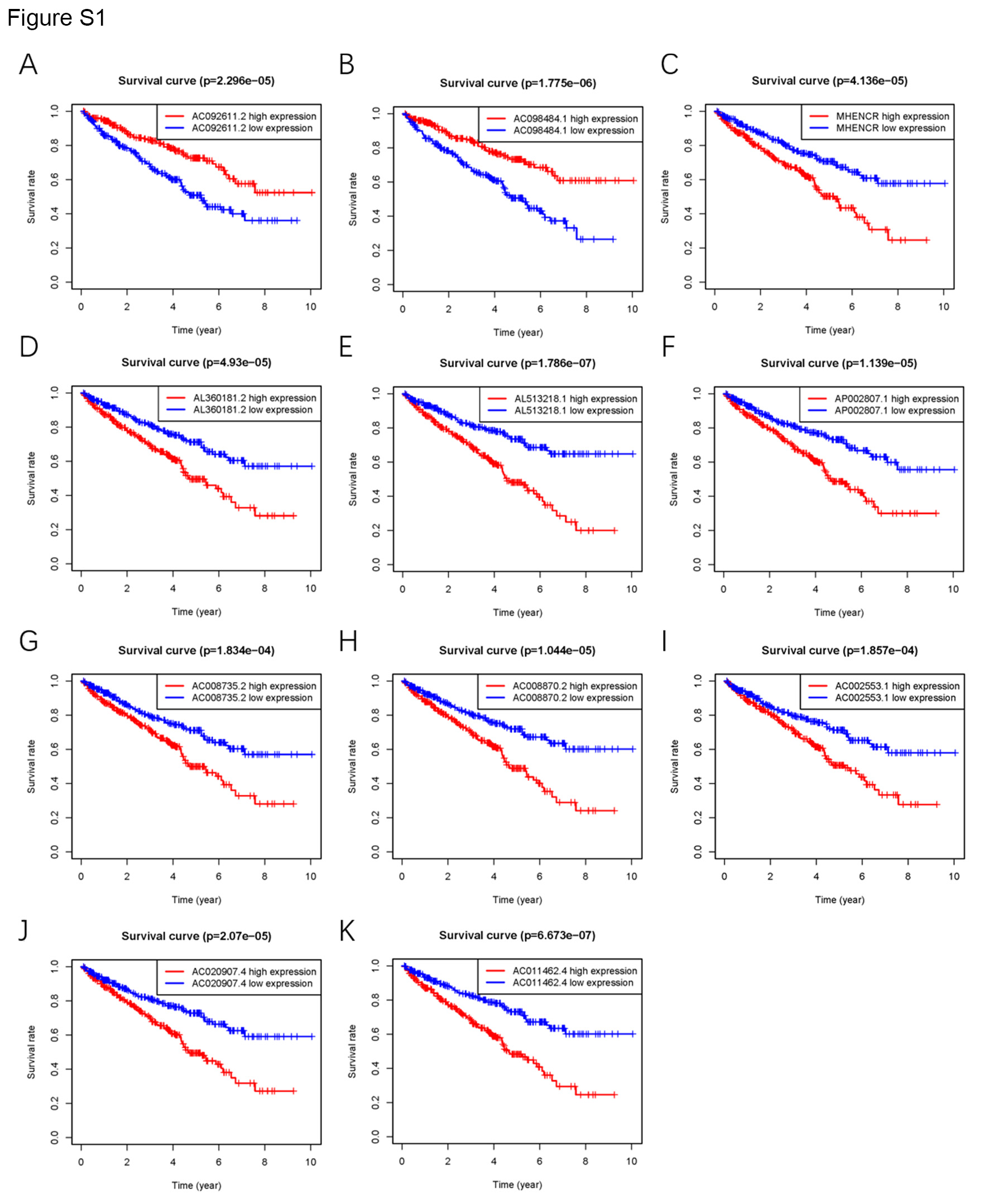

Supplement: Supplementary Figure 1 — The Kaplan-Meier analysis of AC092611.2, AC098484.1, AC002553.1, AL360181.2, AP002807.1, AL513218.1, AC008735.2, MHENCR, AC020907.4, AC011462.4 and AC008870.2. [file Image_1.jpeg]
